# Collectanea Medica

**Published:** 1812-06

**Authors:** 


### COLLECTANEA MEDICA,

CONSISTING OF

ANECDOTES, FACTS, EXTRACTS, ILLUSTRATIONS, QUERIES, SUGGESTIONS, &c.

RELATING TO THE

*History or the Art of Medicine, and the Auxiliary Sciences.*

---

### *Degree of Cold suffered by Reptiles and Insects, without depriving them of Life.*

THE degree of cold sustained by these classes of animals is truly astonishing. Their fluids suffer a perfect congelation into solidity, and yet the vital principle is not destroyed; for, on thawing them with some degree of care, they they recover all their functions. Mr. Hearne, whose researches to the north and north-west of Hudson's Bay, have excited much interest, observes, "that, as the winter approaches, in that climate, the frogs creep under the moss, at a considerable distance from the water, where they remain in a frozen state till spring. I have frequently seen them with the moss frozen as hard as ice, in which state their legs are as easily broken off as a pipe-stem, without the least sensation to the animal; but, by wrapping them up in warm skins, and exposing them to a slow fire, they soon recover life, and the mutilated animal gains its usual activity; but, if they are suffered to freeze again, they are past all recovery. The same may be said of several species of spiders, and all the grub kind, which are very numerous in these parts. I have seen thousands of them dug up with the moss, all of which were inclosed in a thick web, which nature teaches them to spin on these occasions; yet they were, apparently, all frozen as hard as ice. The spiders, if let fall from any height on a hard substance, would rebound like a grey pea; and all the grub kind are so hard frozen, as to be as easily broken as a piece of ice of the same size; yet, when exposed to a slow heat, even in the depth of winter, they will soon come to life, and in a short time recover their usual motions."

This is so extraordinary as almost to pass belief; but it receives a confirmation by the statements of Dr. Lister, in an early volume of the Phil. Trans. This naturalist says, that he saw flies of various sorts frozen so hard as to ring like metal when thrown against glass, and yet they recovered on being thawed.

---

### *Jumping Ague.*

This odd disease, in Scotland, where it has mostly occurred, has been called *the louping* (leaping). The late Lord Monboddo (Ancient Metaphysics) has printed a strongly marked case. I had an opportunity, he says, of being very particularly and accurately informed of the progress of this complaint in one instance. It was the case of a young girl in the neighbourhood of my house in the country. The girl was attacked with this disease in the spring, when she was about sixteen years of age, and it lasted something more than three months. The fit always seized her in the daytime, commonly about seven or eight o'clock in the morning, after she had been out of bed two or three hours. It began with a heaviness and drowsiness, which ended in sleep, for her eyes were close shut. In this condition, she would leap upon stools and tables with great agility; then she would would get out of the cottage where she lived with her father, mother, and brother, and run with great violence, and much faster than she would do when well, but always with a certain destination to some one place in the neighbourhood; and to which place she often said, when she found the fit coming upon her, that she was to go; and, after she had gone to the place of her destination, if she did not then awake, she came back with the same certain direction, though she did not always keep the high road, but frequently went a nearer way across the fields; and, though her road, for this reason, was often very rough, she never fell, notwithstanding the violence with which she ran. But, all the while she ran, her eyes were quite shut, as her brother attests, who often ran with her to take care of her, and who, though he was much older, stronger, and cleverer, than she, was hardly able to keep up with her. When she told, before the fit came on, to what place she was to run, she said she dreamed the night before that she was to run to that place; and, though they sometimes dissuaded her from going to a particular place, as to my house, for example, where they said the dogs would bite her, she said she would run that way, and no other. When she awaked, and came out of her delirium, she found herself extremely weak; but soon recovered her strength, and was nothing worse for it; but, on the contrary, very much the worse for being restrained from running. When she awaked, and came to herself, she had not the least remembrance of what had passed while she was asleep. Sometimes she would run upon the top of the earthen fence which surrounded her father's little garden; and, though the fence was of an irregular figure, and very narrow at top, yet she never fell from it, nor from the top of the house, upon which she would sometimes get by the assistance of this fence, though her eyes were then likewise shut. Some time before the disorder left her, she dreamed, as she said, that the water of a well in the neighbourhood, called the dripping-well, would cure her; and accordingly she drank of it very plentifully, both when she was well and when she was ill. Once, when she was ill, she expressed, by signs, a violent desire to drink of it (for she did not, while in the fit, speak so as to be intelligible), and, they having brought her other water, she would not let it come near her, but rejected it with great signs of aversion; but when they brought her the water of this well, she drank it greedily, her eyes being all the while shut. Before her last fit came upon her, she said she had just three leaps to make, and she would neither leap nor run more. It appears that this occurred as she promised, for the disease returned no more. In her account of the the progress of her feelings as the paroxysm came upon her, she gives a faithful picture of the *aura epileptica*. It began at her feet, and, like a coldness or numbness, crept upwards, till it came to her heart; after which she had no more sense or feeling of the condition she was in. This is an unusual variety of an extraordinary morbid state of the nervous system, for which, perhaps, there is still wanting an appropriate generic term: epilepsy, catalepsy, chorea, hysteria, are other varieties, if they may not all be called species. The method of the cure of this patient seems to indicate that the affection was much influenced by the mental principle.

---

The controversy which once agitated the medical world on the subject of bleeding on the side affected, or on the opposite, has not even yet subsided altogether. As late as the year 1780, a *Dissertatio Medica Inauguralis de Homine dextro et sinistro*, was defended at Leyden by Meinard Simon du Sui, in which were examined the separate affections of the right and left sides of the body. This dissertation is divided into two sections. The first chapter of the first section treats of diseases which affect one side only; the second inquires into the causes of these phenomena; the third discusses the opinion, whether, in pleurisy, and other diseases, bleeding on the affected side, or on that opposite, is to be preferred. The second section also of three chapters examines, first, the nervous affections of one side only; second, details opinions concerning the causes of these affections; and the third examines the validity of the opinions respecting the decussation of nerves. Is this pamphlet, on a subject of considerable interest, any where now to be found?

---

*Experiments on the Strength of Men and Horses in moving Machines.—By* M. Schulze.

Those who have had occasion to construct machines intended to be moved by men or animals, are sufficiently aware how important it is to be acquainted with the quantity of motion that can be attributed to either of them, in order to estimate with accuracy the effect which it is proposed to obtain by the machine. It is well known that the arrangement of the whole depends entirely on the ratio of the velocity of the motive force to the resistance. This was the reason that long ago induced them to take the trouble of determining the strength as well as velocity exerted by men and animals when they are made to move machinery; and the results they obtained, which have been commonly made use of in computing the effect of machines are, that men exert from 27 to 30 lbs. with a velocity of from $1\frac{1}{2}$ to 2 feet per second;

 and and that a horse has about seven times more strength than a man, with a velocity of from 4 to 6 feet per second.

These are the data which we have been obliged to use whenever it became necessary to compute the effect of a machine moved by men or horses. It is evident that the force must be diminished when the velocity is increased, and *vice versâ:* but we are not yet certain of the method of finding the ratio of the diminution or augmentation of this force to the velocity. Euler has given us two different formulæ to compute this ratio; but no one has hitherto attempted to verify by experiment, which of them is to be preferred, although they differ very considerably from each other. If we put $P$ for the absolute force which takes place when we simply consider equilibrium, $C$ the absolute velocity which takes place when the man or animal moves freely and without being overcome by the resistance; $p$ the relative force, and $c$ the corresponding velocity, we have by the first of these formulæ,

$$p = P\left(1 - \frac{c}{C}\right)^2$$

Whereas the second gives us

$$p = P\left(1 - \frac{c^2}{C^2}\right).$$

As I am obliged now more than ever to attend to a number of machines, and to compute their effect, it therefore concerns me very much to know exactly in what manner to estimate, compare, and fix the strength and velocity of men and animals which are used for moving various machines proper for different purposes.

With this view I made with considerable care the experiments I am now about to detail; which of course would have been very expensive, had it not been for some facilities which other persons may not possess.

To make the experiments on human strength, I took promiscuously 20 men of different sizes and constitutions, whom I measured and weighed; the result of which is given in the following Table:

| Order. | Size. | | | Weight. | Order. | Size. | | | Weight. |
|---|---|---|---|---|---|---|---|---|---|
| 1 | 5′ | 3″ | 4‴ | 122 | 11 | 5′ | 9″ | 7‴ | 132 |
| 2 | 5 | 2 | 3 | 134 | 12 | 5 | 1 | 4 | 157 |
| 3 | 5 | 7 | 2 | 165 | 13 | 5 | 3 | 2 | 175 |
| 4 | 5 | 5 | 0 | 131 | 14 | 5 | 4 | 1 | 117 |
| 5 | 5 | 11 | 2 | 177 | 15 | 5 | 10 | 8 | 192 |
| 6 | 6 | 0 | 4 | 158 | 16 | 5 | 0 | 3 | 133 |
| 7 | 5 | 8 | 3 | 180 | 17 | 4 | 11 | 2 | 147 |
| 8 | 5 | 2 | 1 | 117 | 18 | 5 | 3 | 9 | 124 |
| 9 | 5 | 4 | 8 | 140 | 19 | 5 | 6 | 0 | 163 |
| 10 | 5 | 0 | 4 | 126 | 20 | 5 | 10 | 1 | 181 |

To find the strength that each of these men might exert to raise a weight vertically, I made the following experiments:

I took various weights, increasing by 10lbs. from 150lbs. up to 250lbs.; all these weights were of lead, having circular and equal bases. To use them with success in the proposed experiments, I had at the same time a kind of bench made, in the middle of which was a hole of the same size as the base of my weights: this hole was shut by a circular cover when pressed against the bench; at other times it was kept at about the distance of a foot and a half above the bench, by means of a spring and some iron bars. To prevent the weight with which this cover was loaded during the experiment from forcing down the cover lower than the level of the surface of the bench, I had several grooves made in the four iron bars, which sustained the cover, and which at the same time served to hold up the cover at any height where it might arrive by the pressure of the springs as soon as the pressure of the weight ceased.

After having laid the 150lbs. weight on the cover, and the other weights in succession increasing by 10lbs. up to 250lbs. I made the following experiments with the men whose size and weight are given above, by making them lift up the weights as vertically as possible all at once, and by observing the height to which they were able to lift them. The following Table gives the heights observed for the different weights marked at the head of the Table.

| | 150 | | 160 | | 170 | | 180 | | 190 | | 200 | | 210 | | 220 | | 230 | | 240 | | 250 |
|---|---|---|---|---|---|---|---|---|---|---|---|---|---|---|---|---|---|---|---|---|---|
| | ″ | ‴ | ″ | ‴ | ″ | ‴ | ″ | ‴ | ″ | ‴ | ″ | ‴ | ″ | ‴ | | | | | | | |
| 1 | 7 | .9 | 6 | 4 | 4 | 11 | 4 | 4 | 3 | 8 | 2 | 8 | 1 | 1 | | | | | | | |
| 2 | 7 | 10 | 6 | 6 | 5 | 7 | 4 | 7 | 3 | 11 | 2 | 5 | 0 | 5 | ″ | ‴ | ″ | ‴ | | | |
| 3 | 7 | 9 | 7 | 3 | 6 | 5 | 5 | 9 | 4 | 11 | 4 | 0 | 3 | 0 | 1 | 7 | 0 | 3 | ″ | ‴ | |
| 4 | 8 | 3 | 7 | 6 | 7 | 2 | 5 | 10 | 5 | 3 | 4 | 7 | 4 | 0 | 3 | 8 | 3 | 1 | 1 | 4 | |
| 5 | 12 | 4 | 11 | 1 | 9 | 7 | 8 | 5 | 7 | 10 | 7 | 1 | 5 | 10 | 4 | 7 | 3 | 2 | 1 | 3 | |
| 6 | 14 | 5 | 14 | 0 | 13 | 5 | 12 | 8 | 11 | 5 | 10 | 1 | 8 | 6 | 6 | 6 | 4 | 1 | 0 | 1 | |
| 7 | 12 | 11 | 11 | 3 | 10 | 5 | 9 | 3 | 8 | 1 | 6 | 9 | 5 | 3 | 3 | 8 | 1 | 11 | 0 | 2 | |
| 8 | 11 | 9 | 10 | 2 | 9 | 4 | 8 | 11 | 8 | 1 | 6 | 11 | 5 | 10 | 5 | 1 | 3 | 2 | 1 | 0 | |
| 9 | 9 | 5 | 8 | 3 | 7 | 1 | 5 | 6 | 4 | 1 | 2 | 9 | 1 | 3 | | | | | | | |
| 10 | 8 | 1 | 6 | 5 | 4 | 7 | 3 | 9 | 2 | 5 | 1 | 7 | 0 | 4 | | | | | | | |

This Table proves to us that the size of the men employed to raise the weights vertically has considerable influence on the height to which they brought the same weight. We find also by this that the height diminishes in a much more considerable ratio than the weight increases; and we may therefore conclude, that it is advantageous to employ large men when it becomes necessary to draw vertically from from below upwards: and, on the contrary, it is more advantageous to employ men of a considerable weight, when it is required to lift up loads by means of a pulley about which a cord passes, that the workmen draw in a vertical direction, from above downwards. To find the absolute strength of these men in a horizontal direction, I took the following method:

Having fixed over an open pit a brass pulley extremely well made, of 15 inches diameter, whose axis, made of well polished steel to diminish the friction, was ¾ inch in diameter; I passed over this pulley a silk cord worked with care to give it both the necessary strength and flexibility. One of the ends of this cord carried a hook to hang a weight to it which hung vertically in the pit, whilst the other end was held by one of the 20 men, who in the first order of the following experiments made it pass above his shoulders; instead of which, in the second, he simply held it by his hands.

I had taken the precaution to construct this in such a manner that the pulley might be raised or lowered at pleasure, in order to keep the end of the cord held by the man always in a horizontal direction, according as the man was tall or short, and exerted his strength in any given direction.

I had made the necessary arrangements so as to be able to load successively the basin of a balance which I had attached to the hook at the end of the cord which descended into the pit, whilst the man who held the other end of this cord employed all his strength without advancing or receding a single inch.

The following Table gives the weights placed in the basin when the workmen were obliged to give up, having no longer sufficient strength to sustain the pressure occasioned by the weight. To proceed with certainty I increased the weight each time by five pounds, beginning from 60, and I took the precaution to make this augmentation in equal intervals of time, having always precisely a space of 10 seconds between them. The result of these observations repeated, several days in succession, is contained in the following Table.

When the cord passes over the shoulders of the workmen:

| Order. | lbs. | Order. | lbs. | Order. | lbs. | Order. | lbs. |
|---|---|---|---|---|---|---|---|
| 1 | 95 | 6 | 100 | 11 | 95 | 16 | 95 |
| 2 | 105 | 7 | 115 | 12 | 100 | 17 | 100 |
| 3 | 110 | 8 | 105 | 13 | 110 | 18 | 90 |
| 4 | 100 | 9 | 95 | 14 | 90 | 19 | 100 |
| 5 | 105 | 10 | 90 | 15 | 110 | 20 | 105 |

When the cord was simply held before the man:

| Order. | lbs. | Order. | lbs. | Order. | lbs. | Order. | lbs. |
|---|---|---|---|---|---|---|---|
| 1 | 90 | 6 | 100 | 11 | 90 | 16 | 90 |
| 2 | 105 | 7 | 110 | 12 | 90 | 17 | 90 |
| 3 | 105 | 8 | 100 | 13 | 100 | 18 | 85 |
| 4 | 90 | 9 | 90 | 14 | 85 | 19 | 100 |
| 5 | 95 | 10 | 85 | 15 | 105 | 20 | 100 |

These two Tables show that men have less power in drawing a cord before them than when they make it pass over their shoulders: it shows us also that the largest men have not always the greatest strength to hold, or to draw in a horizontal direction, by means of a cord. To obtain the absolute velocity of these 20 men, I proceeded as follows:

Having measured very exactly a distance of 12,000 Rhinland feet in a plain nearly level, I caused these 20 men to march with a good pace, but without running, and so as to continue during the space of four or five hours; the following is the time employed in describing this space, with the velocity resulting for each of them.

| Order | Time. | Veloc. | Order | Time. | Veloc. | Order | Time. | Veloc. |
|---|---|---|---|---|---|---|---|---|
| 1 | 40·18 | 4·94 | 8 | 40· 9 | 4·99 | 15 | 36·17 | 5·51 |
| 2 | 41·12 | 4·85 | 9 | 40·20 | 4·96 | 16 | 41·28 | 4·82 |
| 3 | 39· 8 | 5·55 | 10 | 40·51 | 4·90 | 17 | 42·25 | 4·71 |
| 4 | 39·40 | 5·04 | 11 | 36·17 | 5·51 | 18 | 40·19 | 4·98 |
| 5 | 34·19 | 5·83 | 12 | 38·11 | 5·24 | 19 | 39·57 | 5·01 |
| 6 | 35·11 | 5·68 | 13 | 38· 5 | 5·25 | 20 | 37·51 | 5·29 |
| 7 | 38· 7 | 5·25 | 14 | 37· 1 | 5·40 | | | |

It is necessary to mention, with regard to these experiments, that I took care to place at certain distances persons in whom I could place confidence, in order to observe whether these men marched uniformly and sufficiently quick, without running.

Having thus obtained, not only the absolute force, but the absolute velocity also, of several men, I took the following method to determine their relative force.

I made use of a machine composed of two large cylinders of very hard marble, which turned round a vertical cylinder of wood, and moved by a horse, which described in his march a circle of 10 Rhinland feet. This machine appeared to me the most proper to make the following experiments, which serve to determine the relative strength that the men had employed to move this machine, and which I use hereafter to determine which of Euler's two formulæ ought to be preferred.

To

To obtain this relative force, I took here the same pulley which served me in the preceding experiments, by applying a cord to the vertical cylinder of wood, and attaching to the other end of this cord, which entered into an open pit, a sufficient weight to give successively to the machine different velocities.

Having applied in this manner a weight of 215 lbs. the machine acquired a motion, which, after being reduced to an uniform motion, taking into account the acceleration of the weight, of the friction, and of the stiffness of the cord, gave 2·41 feet velocity; and, having applied in the same manner a weight of 220 lbs. the resulting uniform motion gave a velocity of 2·47 feet. I only mention these two limits because they serve as a comparison with what immediately follows: I began these experiments with a weight of 100 lbs. and increased it by 5 every time from that number up to 400 lbs.

I made this machine move by the seven first of my workmen, placing them in such a way that their direction remained almost perpendicular to the arm on which was attached the cord which passed over their shoulders in an almost horizontal direction.

Thus situated, they made 281 turns with this machine in two hours, which gave for their relative velocity $c = 2·45$ feet per second. We have also the absolute force, or $P$, from these 7 men, by the above Table $= 730$ lbs. and their absolute velocity or $C = 530$ feet.

Therefore, by substituting these values in the first formula, we find the relative force $p = 205$ lbs. which agrees very well with what we have just found above.

If, instead of this first formula, the second be taken, it gives $p = 153$ lbs. which is far too little.

By this it is evident, that the first of Euler's two formulæ is to be preferred in all respects. I have also made a great number of combinations, and I almost always found the same effect.

Dividing the 205 lbs. which we have just found by 7, the number of workmen, we get 29 lbs. for the relative force, with 2·45 feet relative velocity for each man, which is rather more than the values commonly adopted in the computation of machinery. A number of other observations on different machines, which I intend to relate another time, have given me the same result; that is to say, we must value the mean human strength at 29 or 30 lbs. with a velocity of $2\frac{1}{2}$ feet per second.

To obtain the ratio of the strength of a horse to that of a man, I had the same machine moved by a horse without altering any thing; and I found by ten different horses which

 I used

I used successively, that a horse makes 603 turns in 2 hours instead of 281: therefore, by supposing the static motion of a horse 7 times greater than that of a man, we find that the former has 5·3 feet per second of velocity.

By this it is evident, that the effect of a horse is 14 times greater than that of a man, or, which amounts to the same thing, 14 men must be used instead of 1 horse. Hence it appears, that it is much more advantageous to employ horses than men in moving machines, if other reasons did not require us to prefer men.—*Phil. Mag.*

